# Complete sequences of the mitochondrial DNA of the *Grateloupia filicina* (Rhodophyta)

**DOI:** 10.1080/23802359.2017.1419097

**Published:** 2018-01-05

**Authors:** Yue Li, Maria Dyah Nur Meinita, Tao Liu, Shan Chi, Hongxin Yin

**Affiliations:** a College of Marine Life Sciences, Ocean University of China, Qingdao, P. R. China;; b Faculty of Fisheries and Marine Science, Jenderal Soedirman University, Purwokerto, Indonesia;; c Qingdao Haida BlueTek Biotechnology Co., Ltd, Qingdao, P. R. China

**Keywords:** Mitochondrial genome, *Grateloupia filicina*, phylogenetic analysis

## Abstract

In this study, we sequenced and analyzed the complete mitogenome of *Grateloupia filicina* (Lamouroux) C. Agardh. The complete *G. filicina* mitogenome was 29,274-bp long, containing 51 genes, including 24 protein-coding genes, 1 intron, 2 rRNA genes, 24 tRNA genes, and 1 unidentified open reading frame. Twenty-one of the 24 (87.5%) protein-coding genes ended with the stop codon TAA, whereas 3 (12.5%) ended with TAG. All the protein-coding genes in *G. filicina* used the start codon ATG. Phylogenetic analysis revealed that *G. filicina* clustered with *G. taiwanensis.* The complete mitochondrial genome sequence provided here would be useful for understanding the evolution of *Grateloupia* further.

Red alga *Grateloupia filicina* (Lamouroux) C. Agardh, is an edible marine macroalga. It has been widely reported throughout the tropical to warm temperate regions of the world (Wynne 1998; Masuda et al. 2000). It is used as food and as a source of carrageenan (Migita 1988; Nikapitiya et al. 2007). Many studies on this species focus on the improvement of culture techniques (Wong and Chang 2000; Baweja and Sahoo 2009) and phylogenetic analysis (Kawaguchi et al. 2001). However, genomic studies on this species are relatively limited.

Here, we determined the complete mitogenome sequence of *G. filicina.* The genomic DNA of one *G. filicina* individual collected from a population located in eastern China (Xiangshan Harbor, Zhejiang Province, 29°30′20′′N, 121°35′6′′E) was used for genome sequencing. The specimen was deposited in the Culture Collection of Seaweed at the Ocean University of China under the accession number: 2016030029. Paired-end reads were sequenced using the HiSeq × Ten system (Illumina, San Diego, CA, USA). Approximately, 9 Gb of paired-end (150 bp) sequence data was randomly retrieved from the total sequencing output, and used as input into NOVOPlasty (Dierckxsens et al. 2017) to assemble the mitochondrial genome. *G. taiwanensis* (GenBank accession number: KM999231) was used as the seed sequence. Transfer RNA genes were identified using the tRNAscan-SE Search Server (Schattner et al. 2005). Other regions of the mitogenome were annotated by comparing with the mitogenome of *G. taiwanensis* using Geneious R10 (Biomatters Ltd., Auckland, New Zealand). Phylogenetic analysis of a set of 22 conserved protein-coding genes (*atp4*, *atp6*, *atp8, atp9, cob, cox1, cox2, cox3, nad1, nad2, nad3, nad4, nad4L, nad5, nad6, rpl16, rps3, rps11, rps12, sdhB, sdhD,* and *tatC*) present in 14 Rhodymeniophycidae mitogenomes was conducted. The amino acid sequences were aligned using MEGA 5.0 software (Tamura et al. 2011). The concatenated alignments were generated and poorly aligned regions were removed using the Gblocks server (http://phylogeny.lirmm.fr/phylo_cgi/one_task.cgi?task_type=gblocks) (Castresana 2000). Maximum-likelihood (ML) tree search and ML bootstrap analysis were performed using RaxML (Stamatakis 2006). The protein sequence of *Asparagopsis taxiformis* was used as an outgroup.

The complete mitogenome of *G. filicina* (GenBank accession number: MG598532) comprised a circular DNA of 29,274 bp. The overall A-T content of the complete mitogenome was 68.0%. The mitogenome contained a set of 51 genes, including 24 protein-coding genes, 2 rRNA genes, 24 tRNA genes, and 1 unidentified open reading frame (ORF). One intron was inserted in the *cox1* gene. Twenty one of the 24 (87.5%) protein-coding genes had a TAA stop codon, and three (12.5%) had TAG. All the protein-coding genes in *G. filicina* initiated transcription from the start codon ATG. The lengths of two ribosomal RNA genes were 2595 bp (*rnl* rRNA) and 1389 bp (*rns* rRNA). ML analyses showed that *G. filicina* was clustered together with *G. taiwanensis* (Figure 1). The complete mitochondrial genome sequence provided here would be useful for further understanding the evolution of *Grateloupia*.

**Figure 1.**
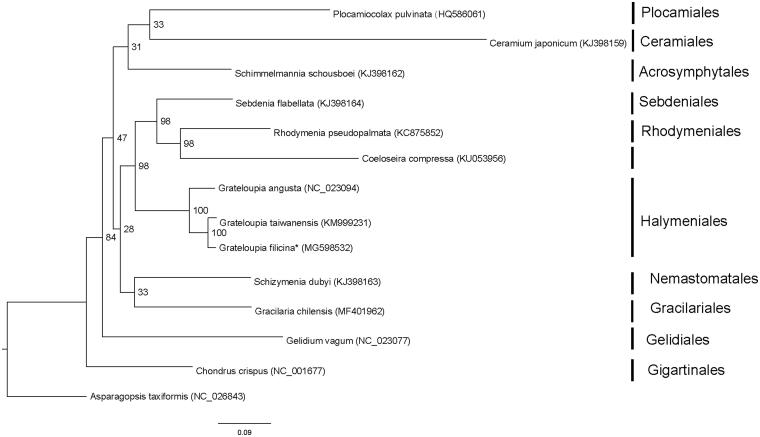
Phylogenetic tree (maximum-likelihood) of 14 representative Rhodymeniophycidae species based on the 22 mitochondrial protein-coding genes. Numbers along branches are RaxML bootstrap supports based on 1000 nreps. Asterisks after species names indicate newly determined mitochondrial genomes.
